# The Effects of Prenatal and Postnatal Exposure to 50-Hz and 3 mT Electromagnetic Field on Rat Testicular Development

**DOI:** 10.3390/medicina59010071

**Published:** 2022-12-29

**Authors:** Nevin Ersoy, Burcu Acikgoz, Ilkay Aksu, Amac Kiray, Husnu Alper Bagriyanik, Muge Kiray

**Affiliations:** 1 Department of Histology&Embryology, Medical Faculty, Dokuz Eylul University, 35330 Izmir, Turkey; 2 Health Sciences Institute, Dokuz Eylul University, 35330 Izmir, Turkey; 3 Izmir Biomedicine and Genom Center, 35330 Izmir, Turkey; 4 Department of Physiology, Medical Faculty, Dokuz Eylul University, 35330 Izmir, Turkey; 5 Department of Anatomy, Medical Faculty, Dokuz Eylul University, 35330 Izmir, Turkey

**Keywords:** electromagnetic field, testis development, rat, the SRC homology 3 (SH3) and multiple Ankyrin repeat domain (SHANK3), insulin-like growth factor-1 (IGF1), vascular endothelial growth factor (VEGF), oxidative stress, hormones

## Abstract

*Background and objectives:* It has been shown that electromagnetic fields (EMFs) have negative effects on the reproductive system. The biological effects of EMF on the male reproductive system are controversial and vary depending on the frequency and exposure time. Although a limited number of studies have focused on the structural and functional effects of EMF, the effects of prenatal and postnatal EMF exposure on testes are not clear. We aimed to investigate the effects of 50-Hz, 3-mT EMF exposure (5 days/wk, 4 h/day) during pre- and postnatal periods on testis development. *Materials and Methods:* Pups from three groups of Sprague-Dawley pregnant rats were used: Sham, EMF-28 (EMF-exposure applied during pregnancy and until postnatal day 28), EMF-42 (EMF-exposure applied during pregnancy and until postnatal day 42). The testis tissues and blood samples of male offspring were collected on the postnatal day 42. *Results:* Morphometric analyses showed a decrease in seminiferous tubule diameter as a result of testicular degeneration in the EMF-42 group. Follicle-stimulating hormone (FSH) and luteinizing hormone (LH) levels were decreased in the EMF-42 group. Lipid peroxidation levels were increased in both EMF groups, while antioxidant levels were decreased only in the EMF-28 group. We found decreased levels of vascular endothelial growth factor (VEGF) and insulin-like growth factor-1 (IGF1) in the EMF-42 group, and decreased levels of the SRC homology 3 (SH3) and multiple ankyrin repeat domain (SHANK3) in the EMF-28 group in the testis tissue. *Conclusions:* EMF exposure during pre- and postnatal periods may cause deterioration in the structure and function of testis and decrease in growing factors that would affect testicular functions in male rat pups. In addition to the oxidative stress observed in testis, decreased SHANK3, VEGF, and IGF1 protein levels suggests that these proteins may be mediators in testis affected by EMF exposure. This study shows that EMF exposure during embryonic development and adolescence can cause apoptosis and structural changes in the testis.

## 1. Introduction

Electromagnetic field (EMF) exposure has increased in recent years due to developing technology and the increase in the use of electrical devices. Electromagnetic fields can be classified according to the wavelengths. Radio-frequency radiation (RF) (3 MHz to 300 GHz) and extremely low-frequency electromagnetic field (ELF-EMF) (3–300 Hz) are the most common EMF sources. The ELF-EMF is emitted from the electric power network (50 or 60 Hz) and by home appliances. It is recommended to reduce the ELF-EMF exposure as low as possible in areas where people spend more than 4 h per day, since ELF-EMF effects are known to increase with field strength, proximity to source, and duration of exposure [1]. The biological effects of low frequency magnetic field or radio frequency radiation on tissues vary depending on its frequency and exposure time [2,3]. One of the mechanisms explaining the effects of EMF on biological systems is that prolonging the life of free radicals in tissues. High levels of intensity or long-term exposure to EMF increase free radical formation and can lead cell damage. Free oxygen radicals have been implicated in the oxidative damage of biomolecules, such as nucleic acids, proteins, and lipids in tissues [4]. Malondialdehyde (MDA) is the breakdown product of the lipid peroxidation and is known as an oxidative stress marker. There are endogenous antioxidant defense systems against free radicals, such as enzymatic superoxide dismutase (SOD) or glutathione peroxidase (GPx) and non-enzymatic glutathione (GSH) in the organism [5]. EMF could increase lipid peroxidation in different tissues including rat testis [6,7,8]. The male reproductive system is vulnerable to exogenous factors, such as EMF, which can cause reproductive disorders [9]. There are several reports focused on adult rats showing that EMF exposure causes oxidative stress and reduces antioxidant enzyme activities in rat testes [9,10,11]. Studies examining the effects of pre- and postnatal EMF exposure on testicular oxidative stress are limited [12,13].

It has been shown that EMFs have negative effects on the human reproductive system by deteriorating the spermatozoa structure and motility depending on the exposure time [14,15]. Studies also in male rats show that EMF may affect fertility negatively and cause functional and structural changes in the reproductive system [11,16,17]. EMF exposure can cause structural deterioration in the seminiferous tubules of the testis, such as a decrease in epithelial height and diameter [9,18]. Sex hormones are important for maintaining spermatogenesis and the development of reproductive system organs [19]. In males, follicle-stimulating hormone (FSH) initiates the spermatogenesis and luteinizing hormone (LH) promotes androgen production by Leydig cells [20]. The synergistic actions of FSH, LH, and testosterone regulate male fertility [21,22]. EMF exposure may affect fertility negatively by impairing the balance of sex hormones [21].

Insulin-like growth factor-1 (IGF1) is a polypeptide hormone produced in the testis as well as the liver in response to growth hormone (GH) stimulation. IGF1 is important for a healthy onset of puberty and sexual development [23]. The critical role of IGF1 has emerged from studies in knockout mice [24]. It was determined that testicular sizes and body weights decreased in these mice with the IGF1 gene silenced. IGF1 deficient mice also have low sperm count [25]. IGF1 also has an anti-apoptotic effect [26], and high IGF1 levels may play a role in increasing spermatogenesis [27,28]. Vascular endothelial growth factor (VEGF), another growth factor produced in the male reproductive system, is the most important regulator of endothelial growth and permeability [29]. VEGF is synthesized from Sertoli and Leydig cells in the male genital tract, as well as from peritubular cells of the epididymis, prostate epithelial cells, and seminal vesicle. VEGF is very important in germ cell homeostasis and may regulate male fertility in testes [29,30,31].

The SRC homology 3 (SH3) and multiple ankyrin repeat domain (SHANK) proteins are scaffold proteins of the postsynaptic density that contribute to the molecular composition and integrity of glutamatergic synapses by interacting with ion channels, scaffold proteins, receptors, and cytoskeletal proteins [32]. SHANK proteins are predominantly found in the brain, since they serve as major functional and structural scaffolding proteins and regulate other postsynaptic density proteins in the presynapse via the neuroligin-neurexin transsynaptic complex [33]. While SHANK1 is expressed only in the brain, SHANK2 and SHANK3 are also expressed in kidneys, liver, testicles, pancreas, spleen, and heart [34]. It is thought that SHANK2 expressed in endocrine organs may be associated with microfilament-dependent control of secretory activity [35]. Although the possible role of these proteins in the rat reproductive system has been shown, there is not enough information about their function and their interaction with the cytoskeleton and cell membrane in the reproductive system. While it is known that environmental factors during development may cause deterioration in the synaptic complex by affecting SHANK3, it is not known whether it has an effect on the testis.

Limited studies in the literature examined the changes due to EMF in developing testicular tissue, but no study was conducted on the relationship with SHANK3 protein, IGF1, and VEGF [16,36]. We aimed to investigate the effects of exposure to EMF in pre- and postnatal periods on testicular development by biochemical and histomorphological evaluations and physiological role of SHANK3, IGF1 and VEGF in male offspring.

## 2. Materials and Methods

### 2.1. Animals

**Figure 1:**
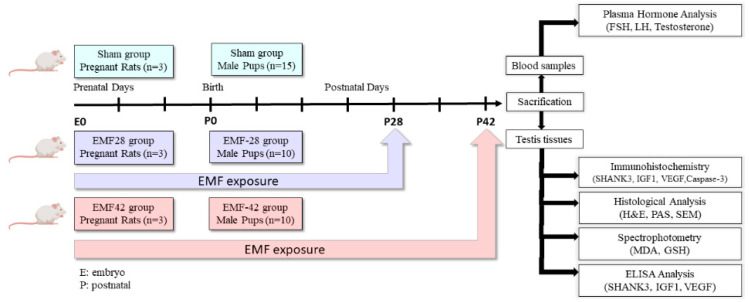
The scheme of the experiment. EMF, electromagnetic field; FSH, follicle-stimulating hormone; LH, luteinizing hormone; SHANK3, SH3 and multiple ankyrin repeat domain; IGF1, insulin-like growth factor-1; VEGF, vascular endothelial growth factor; H&E, hematoxylin-eosin; PAS, periodic acid-Schiff; SEM, scanning electron microscopy; MDA, malondialdehyde; GSH, glutathione.

This study was approved by the Animal Research Ethics Committee of the Dokuz Eylul University Medical Faculty, Turkey (approval number: 26/2017, date: 14 December 2017). All experiments were performed in accordance with the guidelines of the Declaration of Helsinki. The rats were left under controlled laboratory conditions to acclimatize for one week. After the one-week acclimatization period, the vaginal smears were examined in the morning with a light microscope to determine the estrous phase of the female Sprague-Dawley rats (200–250 g). Selected female rats were kept with male rats as pairs in individual cages overnight. As the evidence of mating, the vaginal plug was checked in the next morning and considered as day 0 of pregnancy [37]. Pregnant rats were fed with food and water ad libitum and housed in individual polycarbonate cages until birth. Three pregnant rats were randomly selected as a Sham group while six pregnant rats were exposed to EMF (3 mT, 4 h/day) during gestation. Rat pups were kept with their mothers after parturition and fed with breast milk until postnatal day 21. After weaning, pups were fed with standard food and water ad libitum. The EMF-exposed litters were further exposed to EMF (3 mT, 4 h/day, 5 days/week) until postnatal day 28 and divided into two groups on day 28. One group continued to be further exposed to EMF until the 42nd day. The exposure of the second group was terminated on day 28 and they were kept without exposure until the 42nd day. The total exposure time of pups was 9 and 7 weeks respectively. The same conditions, but not the exposure, were applied to the Sham group. Male pups were selected randomly from Sham (*n* = 15), EMF-28 exposed (*n* = 10), and EMF-42 exposed (*n* = 10) groups and sacrificed on postnatal day 42. Blood samples were collected, and testis tissue samples were dissected for further evaluation (Figure 1).

### 2.2. EMF Exposure System

**Figure 2:**
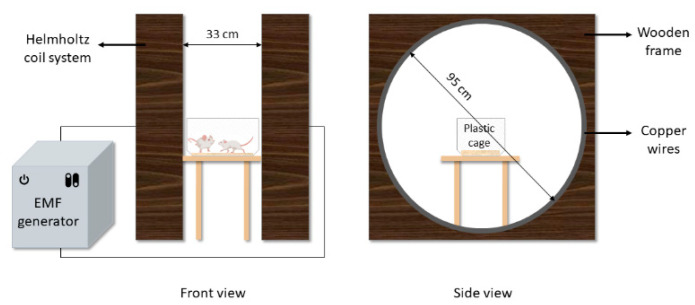
EMF exposure system. EMF; electromagnetic field.

The EMF exposure was applied with a system previously used in our laboratory [38]. EMF of 3 mT was produced with a Helmholtz coil pair system (95 cm in diameter), and the turns were 320 per meter. A copper wire with 2.5 mm diameter was used, and the material of the circle containing the coils was wooden. The two coils are placed at a distance of 33 cm from each other. The coils were connected in series to an alternating current (AC) generator whose output current was 6.43 A at 50 Hz. The magnetic flux density was measured using a digital EMF measuring device, with a Tesla meter accuracy of ±2% (FW Bell, 5170, Orlando, USA). The rats were placed in their plastic cages during the exposure (Figure 2).

### 2.3. Biochemical Analyses

The blood and right testis tissue samples were used for biochemical analysis. The right testis tissues were homogenized in 0.15 M KCl (10%, *w/v*, 104,936, Merck, Germany) using an ultrasonic homogenizer (Bandelin Sonopuls, Germany). The homogenates were centrifuged at 10,000× *g* for 20 min at 4 °C. The supernatant was stored at −80 °C. MDA levels of testis tissues were measured spectrophotometrically using the commercial kit Bioxytech MDA-586 (OxisResearch, Oxis International, Inc., Portland, OR, USA). The detection method is based on the reaction of MDA with a chromogenic reagent at 45 °C. MDA values were determined according to the standard curve formed by measuring absorbance at 586 nm. The measurement of the tissue GSH levels was done by spectrophotometer (T80 PG, instruments, Lecihestershire, UK) using the GSH-420 commercial kit (OxisResearch, USA). The detection method is based on the formation of chromophoric thione. Adding a reducing agent to the buffer-mixed supernatant, the oxidizing glutathione is converted into a reduced form. Chromophoric thione was formed by adding chromogen and increasing the pH value. The absorbance levels were measured at 420 nm and the GSH concentrations were calculated according to the standard curve. The MDA and GSH results were shown as µM/mg protein. Tissue protein levels were measured with Pierce BCA Protein Assay Kit (23227, Thermo Scientific, IL, Waltham, USA). Tissue SHANK3, IGF1, and VEGF analysis were evaluated using the ELISA method, utilizing the ELISA reader BioTek ELx800 (Santa Clara, CA, USA) in accordance with the operating instructions of assay kits (MBS9905957, MyBioSource (San Diego, CA, USA); EK0377, Boster Bio., (Pleasanton, CA, USA), EK0540, Boster Bio., (Pleasanton, CA, USA), respectively). FSH and LH levels in plasma were measured using commercial ELISA kits (Cat. No: 201-11-0183, SunRed, China and Cat. No: 201-11-0180, SunRed, (Shanghai, China), respectively). Plasma total testosterone levels were measured by chemiluminometric immunoassay, which uses advanced acridinium ester, on the ADVIA Centaur XPT analyzer (Siemens Healthineers, Erlangen, Germany).

### 2.4. Histological Analysis

The left testis tissues were fixed in 10% buffered formalin solution for 48 h and embedded in paraffin. Serial sections (5 µm thickness) were taken from the paraffin blocks with a microtome (Thermo Finesse M+, Thermo Scientific). The hematoxylin-eosin (H&E, 05-06002L&05-10007L, (Bio-optica, Milano, MI, Italy) and periodic acid-Schiff (PAS, ab150680, Abcam, Cambridge, UK) stains were used to examine the sections with an image analysis system (Image Focus4 software, The Netherlands) consisting of a light microscope (Euromex Trino-Ox3035, Arnhem, The Netherlands) and a video camera (Oxion HDMI High Definition Colour Camera-VC3036, Arnhem, The Netherlands).

### 2.5. Examination of Spermatogenesis

We used the Johnsen scoring criteria to classify spermatogenesis and assess histological damage in testis under light microscopy. This classification is from 1 to 10 as follows: grade 1—no germ cells and no Sertoli cells in the tubules; grade 2—no germ cells but only Sertoli cells present in the tubules; grade 3—only spermatogonia cell present; grade 4—few spermatocytes present; grade 5—many spermatocytes present; grade 6—few early spermatids present; grade 7—no late spermatids but many early spermatids; grade 8—few (less than five) spermatozoa present per tubule; grade 9—slightly impaired spermatogenesis and many spermatozoa present; grade 10—indicates full spermatogenesis. Randomly 100 round shaped seminiferous tubules were selected from all groups in the experiment and were given a score from 1 to 10 [39].

### 2.6. Measurement of Seminiferous Tubule Diameter (STD) and Basement Membrane Thickness (STBM)

In each H&E-stained section, the 100 most circular seminiferous tubules were randomly identified. STD was measured by Image J software, version 1.53k (National Institutes of Health, Bethesda, USA) using the X20 magnification. STBM was measured on 50 randomly selected seminiferous tubule basement membranes from 3 randomly taken sections stained with PAS and averaged.

### 2.7. Immunohistochemistry

Immunohistochemical analysis was done using SHANK3, IGF1, VEGF, and caspase-3 antibodies to examine the immunoreactivity of seminiferous tubules in the testis. The paraffin sections were deparaffinized overnight at 60 °C, and then in xylol, rehydrated with graded alcohols. The histological slices were incubated with proteolytic enzyme at 37 °C (Digest-All 4, 00–3011, Invitrogen, Carlsbad, USA) for 15 min. Endogenous peroxidase activity was blocked in 3% hydrogen peroxide for 5 min. After washing the slides with phosphate buffer solution, blocking reagent was applied (ScyTek, SensiTek HRP Anti-Polyvalent, Utah, USA) for 20 min at room temperature. After overnight incubation of rat specific antibodies (SHANK 3 (bs 12143R, 1:100, Bioss, Woburn, USA), VEGF (bs 1313R, 1:100, Bioss, USA), IGF1 (bs-0014R, 1:100, Bioss USA), and caspase-3 (AB 3623, 1:50, Millipore, Burlington, USA) at 4 °C, sections were soaked in biotinylated secondary antibody solution for 10 min. Enzyme-labeled (peroxidase) secondary antibody was bound with avidin-biotin complex (streptavidin) solution. The reaction product was then detected with 0.02% diaminobenzidine (DAB, 11718096001, Roche, Germany). For counterstaining, Mayer’s hematoxylin was used. Negative controls were obtained by the exclusion of the primary antibody. The positive immunostaining of the slides was evaluated, and the intensity of the pixels was calculated using ImageJ/FIJI software. The morphometric assessment used seven rats per group. After immunostaining with anti-SHANK-3, anti-VEGF, anti-IGF-1, and anti-caspase-3, 100 seminiferous tubules in the well-defined cross sections were selected per rat in all the experimental groups. As a first step, using ImageJ/FIJI software, we used the color-deconvolution technique to RGB images through H DAB matrices. On DAB matrices, the images were converted to 8-bit, and the threshold was adjusted for DAB detection according to intensity. The pixel intensities of DAB images range from 0 to 255. Value 0 represents the darkest shade of the color while 255 represent the lightest shade of the color in the images as standard. In the histogram profile, we categorized pixel intensity ranges from 0–60 for a score value of 3+, 61–120 for 2+, 121–170 for 1+ and 171–255 for 0 (no staining). The threshold parameters were preserved constantly for all images [40].

### 2.8. Scanning Electron Microscopy (SEM) Evaluation

Testicular tissue sections were fixed with 2.5% gluteraldehyde in phosphate buffer (Electron Microscopy Sciences, 16210, Pennsylvania, USA). After, the samples were washed and post-fixed with 2% osmium tetroxide (Electron Microscopy Sciences, 19110, USA) and then dehydrated. The samples were mounted on an aluminum stub and then coated with 5 nm thickness gold. The prepared samples of testis were examined under scanning electron microscopy. Three-dimensional surface scanning was done with high-definition backscatter detector (HDBSD). Seminiferous tubules ultrastructure was evaluated by using scanning electron microscope (ZEISS Sigma 500, Jena, Germany).

### 2.9. Statistical Analysis

All data were shown as mean ± standard error of the mean (SEM). Data analyses were carried out using the SPSS 24.0 software (IBM Inc., IL, USA). One-way analysis of variance (ANOVA) post-hoc LSD test was used to detect the differences between the groups for normally distributed data. Data were analyzed by using the Levene test and the Shapiro–Wilk test for equality of variances and normal distribution, respectively. Organ weights were analyzed using ANOVA as above and by analysis of covariance (ANCOVA) using final body weight as covariate. The values of *p* < 0.05 were considered statistically significant.

## 3. Results

### 3.1. Body and Testis Weights

There was a significant difference in the mean body weights of rat pups between the Sham and EMF groups (F (2, 32) = 5.97, *p* < 0.05). The mean body weights of EMF-42 group were significantly lower than the Sham and EMF-28 groups (*p* = 0.002 and *p* = 0.013, respectively, Table 1). Estimated marginal means of testis weights after adjustment for final body weight using ANCOVA showed a significant main effect of exposure time with EMF-42 rats having lower testis weight compared to EMF-28 rats (F (2, 32) = 3.79, *p* = 0.033). The difference in the mean testis weights between the Sham and EMF groups was significant (F (2, 32) = 9.88, *p* < 0.05). The mean testis weights of the EMF-42 group were decreased compared to the Sham and EMF-28 groups (*p* < 0.001 and *p* = 0.020, respectively, Table 1).

### 3.2. Biochemical Analyses

The sex hormone levels are shown in Table 2. Plasma FSH levels were significantly different between the groups (F (2, 27) = 3.93, *p* < 0.05). FSH levels decreased in the EMF-42 group compared to Sham and EMF-28 groups (*p* = 0.037 and *p* = 0.015, respectively). There was a significant difference in plasma LH levels between the groups (F (2, 27) = 5.77, *p* < 0.05). LH levels were decreased in the EMF-42 group compared to the Sham and EMF-28 groups (*p* = 0.019 and *p* = 0.003, respectively). There was no significant difference in plasma testosterone levels between groups (*p* > 0.05).

There was a difference in the testis MDA levels between groups (F (2, 29) = 5.44, *p* < 0.05). In the EMF-28 and -42 groups, MDA levels of testis tissues were increased compared to the Sham group (*p* = 0.014 and *p* = 0.006, respectively, Table 3).

A significant difference was found in GSH levels in testis tissue between groups (F (2, 32) = 10.69, *p* < 0.001). Decreased GSH levels of the testis tissue were found in the EMF-28 group compared to the Sham and EMF-42 groups (*p* < 0.001 and *p* = 0.001, respectively, Table 3).

There was a difference in SHANK3 levels in testis tissue between groups (F (2, 15) = 8.10, *p* < 0.05). The SHANK3 levels of the EMF-28 group in testis tissues were found to be lower than the Sham and EMF-42 groups (*p* = 0.013 and *p* = 0.001, respectively, Table 4). In the EMF-42 group, IGF1 levels of testis tissue were found to be lower than the Sham group (F (2, 32) = 5.23, *p* = 0.003). No significant difference was found in IGF1 levels between Sham and EMF-28 groups (*p* > 0.005, Table 4). VEGF levels of the EMF-42 group in testis tissues were significantly lower than the Sham group (F (2, 32) = 4.03, *p* = 0.009). No difference was found in VEGF levels between Sham and EMF-28 groups (*p* > 0.005, Table 4).

### 3.3. Histological Analysis

**Figure 3:**
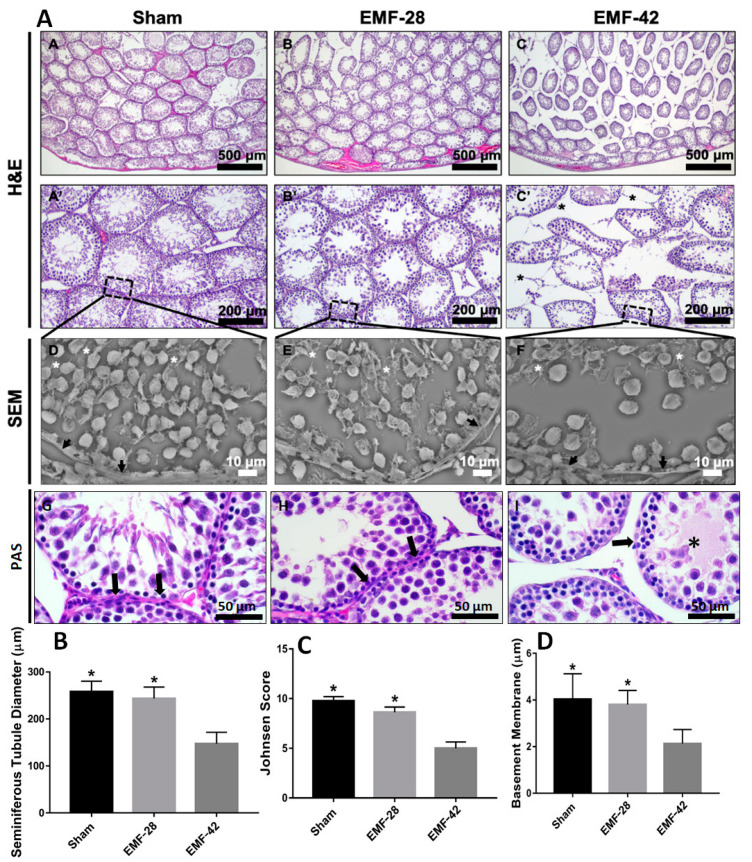
(**A**). Representative photomicrographs of rat testis sections. The top two lines, H&E-stained sections of groups (A-A’, Sham group; B-B’, EMF-28 group; C-C’, EMF-42 group). Third line, scanning electron micrograph (SEM) of testicular tissue of groups (D, Sham; E, EMF-28 group; F, EMF-42 group). Black asterisk, interstitial space between seminiferous tubules, white asterisk, late spermatids; black arrow, seminiferous tubule basement membrane. Bottom line, periodic acid-Schiff (PAS) stained sections in the testis of rats. A, Sham group (*n =* 15); B, EMF-28 (*n =* 10) group; C, EMF-42 (*n =* 10) group. Asterisk, cellular debris; black arrow, seminiferous tubule basement membrane. Scale bar, 50 µm. (**B**,**C**) Morphological parameters of Sham (*n =* 15), EMF-28 (*n =* 10), and EMF-42 (*n =* 10) groups. * Significantly different from EMF-42 group (*p* < 0.0001, one-way ANOVA with subsequent LSD multiple comparisons test). (**D**) Seminiferous tubule basement membrane thickness. * Significantly different from EMF-42 group (*p* < 0.0001, (one-way ANOVA with subsequent LSD multiple comparisons test). EMF: 50 Hz electromagnetic field exposure (4 h/day, 5 days/week for 7 (EMF-28) and 9 (EMF-42) weeks), Sham group was exposed to the same conditions as the EMF group, except for the EMF exposure.

In H&E stained sections, the testis tissue in the Sham group was covered by connective tissue (albugineous tissue) and the complete seminiferous tubule cell series was present. Interstitial tissue was made of connective tissue having Leydig cells and blood vessels. In addition, the seminiferous tubule cell lines were observed to be normal (Figure 3A, A-A’). It was determined that the number of spermatogenic cells in the EMF-42 group decreased compared to the EMF-28 group. There was also an improvement in the seminiferous tubule structure in the EMF-28 group (Figure 3A, B-B’). The seminiferous tubule structure was disrupted, degenerated, and irregularly shaped in the EMF-42 group rats along with a considerable decrease in the spermatogenic cell series. The increased connective tissue between the seminiferous tubules and cell debris accumulated in the seminiferous tubule lumen were found (Figure 3A, C-C’).

The three-dimensional surface scan (SEM-HDBSD) analyses of Sham group testis tissue illustrated the cross section of seminiferous tubules. Seminiferous tubule basement membrane was of regular shape and normal thickness. Spermatogonia, spermatocyte, and spermatid cells were detected in seminiferous epithelium from basal part of the tubule to the lumen (Figure 3A,D). In EMF-28 group, the basement membrane of the seminiferous tubule was uniformly shaped and nearly normal in thickness, and resembled that of the Sham group. Some spermatid cells were observed in the tubule lumens (Figure 3A,E). In EMF-42 group, germ cells and adluminal compartments showed irregular arrangement boundary tissue and wide interstitial spaces in between. In the seminiferous epithelium, the number of spermatogonial series cells decreased compared to Sham and EMF-28 groups (Figure 3A,F).

STD and Johnsen scores are shown in Figure 3B and 3C. STD was decreased in EMF-42 group compared to the Sham and EMF-28 groups (F (2, 750) = 1586, *p* < 0.0001). Johnsen scores of the EMF-42 group were lower than the Sham and EMF-28 groups (F (2, 294) = 1851, *p* < 0.0001).

In PAS stained sections, the seminiferous tubule basement membrane thickness was decreased in EMF-42 group rats compared with Sham and EMF-28 group rats (F (2, 117) = 66.58, *p* < 0.0001). No difference was found between Sham and EMA-28 groups (*p* > 0.05, Figure 3A,D).

### 3.4. Immunohistochemistry

**Figure 4:**
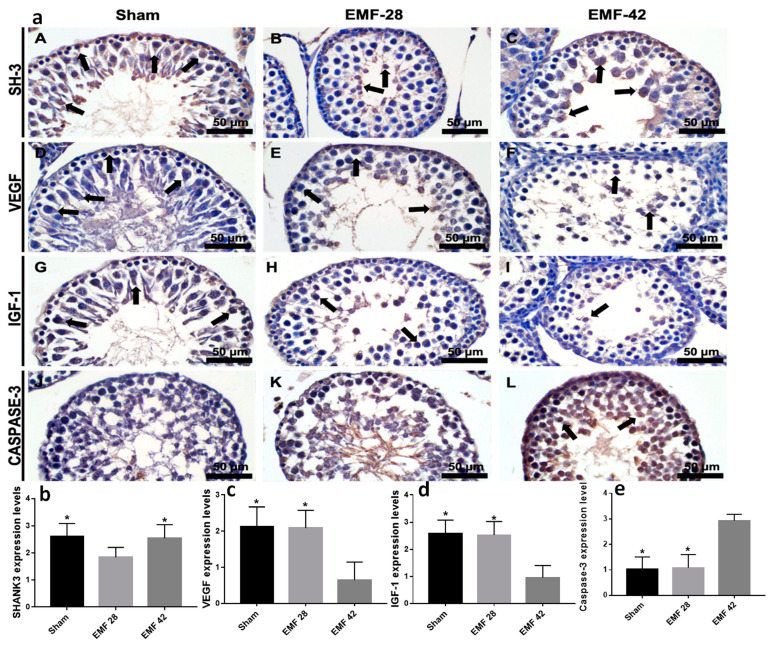
(**a**); Immunostaining of rat testes with SHANK3, VEGF, IGF1, and caspase-3 antibodies. (A, D, G, J—Sham group; B, E, H, K—EMF-28 group; C, F, I, L—EMF-42 group). Black arrows, immunopositive cells. EMF, 50 Hz electromagnetic field exposure (4 h/day, 5 days/week for 7 (EMF-28) and 9 (EMF-42) weeks); Sham group was exposed to the same conditions as the EMF group, except for the EMF exposure. Scale bar, 50 µm. SHANK3, VEGF, IGF1, and caspase-3 immunohistochemistry scores of Sham (*n* = 7), EMF-28 (*n* = 7), and EMF-42 (*n* = 7) group rat pups’ testes (one-way ANOVA with subsequent LSD multiple comparisons test). * *p* < 0.0001, significantly different from EMF-28 group (**b**), * *p* < 0.0001, significantly different from EMF-42 group (**c**–**e**).

SHANK-3, VEGF, IGF1 and Caspase-3 immunoreactivities are shown in Figure 4. There was significantly difference in SHANK-3 immunoreactivity between groups (F (2, 20) = 609.3, *p* < 0.0001). SHANK-3 immunoreactivity was decreased in EMF-28 group compared to the Sham and EMF-42 groups (*p* < 0.0001, Figure 4a,b). No difference was found between Sham and EMF-42 groups (*p* > 0.05).

There was a difference in VEGF and IGF1 immunoreactivities between groups (F (2, 20) = 1905.0, *p* < 0.0001, and F (2, 20) = 2598.0, *p* < 0.0001, respectively). The VEGF and IGF1 immunoreactivities were significantly decreased in EMF-42 group testis tissues compared to Sham and EMF-28 groups (*p* < 0.0001, Figure 4a,c–d). No significant difference was found in VEGF and IGF1 immunoreactivities between the Sham and EMF-28 groups (*p* > 0.05).

Caspase-3 immunoreactivity was increased in EMF-42 group compared to Sham and EMF-28 groups (F (2, 20) = 4330.0, *p* < 0.0001). There was no difference in xaspase-3 immunoreactivity between Sham and EMF-28 groups (*p* > 0.05, Figure 4a,e).

**Figure 5:**
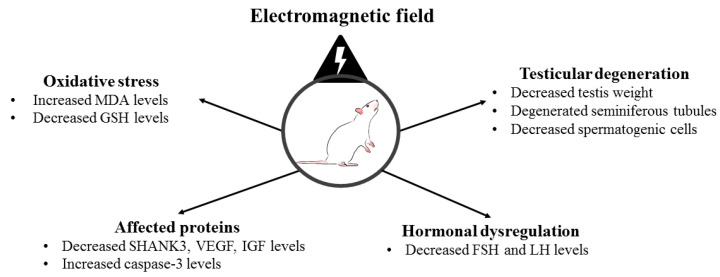
The main findings of the study.

The main findings of the study are summarized in Figure 5.

## 4. Discussion

In this study, we found that early exposure to EMF may cause biochemical, hormonal, and morphological changes in adolescence. This is the first study evaluating the effect of prenatal and postnatal EMF exposure on testis VEGF levels, to our knowledge. Also, this is the first study investigating the effects of EMF and the mediating role of SHANK3 in developing male offspring.

There is no other study showing the oxidative effects of EMF applied in both pre- and postnatal periods on testicular tissue, to our knowledge. Malondialdehyde (MDA) is the breakdown product of the lipid peroxidation and is known as an oxidative stress marker. There are endogenous antioxidant defense systems against free radicals, such as enzymatic superoxide dismutase (SOD) or glutathione peroxidase (GPx) and non-enzymatic glutathione (GSH) in the organism [5]. We found that MDA levels in EMF-28 and EMF-42 groups increased compared to the Sham group. This result shows that EMF exposure we applied causes oxidative damage in testis tissue in adolescence. MDA, as a biomarker of testicular oxidative damage due to EMF, has been examined in adult male rats [9,41,42]. In many studies evaluating the effects of EMF on the rodent reproductive system, different EMF sources have been used and various results have been obtained regarding oxidative stress parameters [43,44,45]. Although most of the studies have found that induced oxidative stress in testis tissue due to EMF exposure [5,17,44,45,46,47], there are some studies showing that EMF exposure does not cause oxidative stress [13,43]. In our study, we found increased MDA levels and decreased GSH levels in the testis tissues due to 50 Hz EMF exposure. In a similar study, it was found that 50 Hz EMF exposure for 42 days caused an increase in testis MDA levels and a decrease in serum total antioxidant capacity, resulting in apoptosis in Sertoli cells in rats [17].

In a study, in which 900 MHz EMF was applied between 13–21 days of pregnancy, the effects of EMF on testicular tissue of 21-day-old pups have examined [45]. They have found increased MDA and apoptosis levels in testis, besides tissue damage in testis, but not a significant difference between the testis weights. Based on these results, they pointed out that the negative effects of prenatal EMF exposure on rat testes may persist after birth, and that oxidative stress associated with EMF might be important in terms of sperm damage and infertility [45]. In a follow-up study, 900 MHz EMF exposure was applied on days 13–21 of pregnancy and its effects on testicular tissue in 60-day-old pups were examined [13]. They have found that EMF may cause decreased tissue weight, increased tissue damage, and induced apoptosis in testis, but no difference in testis MDA levels. They stated that the lack of difference in MDA levels might be caused by the fact that the EMF was applied during the prenatal period, while the measurement was made on the postnatal day 60 [13]. In a study evaluating the effects of 900 MHz exposure applied between postnatal days 21–59, it was found a decrease in tissue weight, an increase in the apoptotic index, and tissue damage in the testis [5]. They also found increased levels of MDA in testis tissue, but no difference was found in GSH levels. They have stated that the EMF exposure during adolescence can lead to changes in oxidative stress biomarkers and the morphology of the rat testis [5]. In our study, we applied 50 Hz EMF exposure during the pre- and postnatal periods. Although there are some differences in experimental procedures and how the EMF was applied, we found increased testis MDA levels in both EMF exposure groups. Interestingly, in our study, endogenous antioxidant parameter GSH levels were decreased only in the EMF-28 group, but not in the EMF-42 group. The decrease in GSH levels in this group can be explained by its use in the reduction reaction of hydrogen peroxide. These results suggest that the protective effects of the antioxidant system against EMF may increase over time.

Sex hormones are important for maintaining spermatogenesis and the development of reproductive system organs [19]. We found that FSH and LH levels decreased in the EMF-42 group compared to the Sham group, but not in the EMF-28 group, while testosterone levels remained unchanged. Decreased FSH and LH levels in the EMF-42 group suggest that long-term EMF exposure affects the central nervous system. In the EMF-28 group, post-exposure period without exposure resulted in recovery in hormonal levels. The effect of EMF on gonadotropic hormones were found to be controversial. Sepehrimanesh et al. have found increased FSH and LH levels and decreased testosterone levels in adult male rats after 900 MHz EMF (4 h/day) exposure for 30 days [21]. On the contrary, decreased FSH, LH, and testosterone levels were found in adult male rats after 2100 MHz EMF (2 h per day) exposure for 16 days [48], whereas Çetkin et al. showed long-term mobile phone exposure caused no change in hormonal levels [49]. Due to the results varying according to the type and duration of EMF exposure, further experimental studies are essential to examine the effect of EMF on hormonal changes in the male reproductive system.

We found a decrease in seminiferous tubule diameters, atrophy in seminiferous tubule epithelial cells, and a decrease in spermatogenic cell lines in the testicles of EMF-42 group rats. It was determined that there was also a decrease in the thickness of the basement membrane of the seminiferous tubule. Likewise, scanning electron microscopy results showed that there was testicular degeneration. It was found that pre- or postnatal exposure to EMF cause degeneration in the seminiferous tubules in previous studies [13,16]. Another study showed that the boundary tissue of the seminiferous tubules was found disrupted at several places and revealed large spaces in the connective tissue [50]. In a study, when the testicular tissue of rats, which were exposed to a mobile phone for 5 months, was examined by SEM, it was observed that there was a decrease in the germ cell population and sperm count, and separation in the seminiferous tubule epithelium and the basal compartments [51]. Another finding in this study is an increase in apoptotic cell density in germinal cells. It is known that EMF has toxic effects on various cells in rats, including germ cells. It has been shown that spermatogenic cell lines in the seminiferous tubule undergo apoptosis and decrease in seminiferous tubule diameter, depending on the electromagnetic field dose and duration [9,48].

In our study, decreased levels and immunoreactivity of IGF1 in testis tissues were found in the EMF-42 group. The IGF system plays an important role in the proliferation and differentiation of Sertoli cells and germ cells in testicular development during embryogenesis [52]. Since FSH is associated with IGF pathways by way of common downstream signaling pathways or FSH-dependent secretion of IGFs, it has been suggested that the IGF system may regulate FSH activity in the gonads [53]. It can be thought that the deterioration may be caused by the decrease in IGF1 levels, due to the decreased FSH levels in the EMF-42 group. In a study examining the effects of 50 Hz electric field applied in the prenatal or postnatal periods on the development of female rats, decreased serum IGF1 levels were found in the prenatal exposure group, but not in the postnatal exposure group [54]. On the other hand, in the study examining the effects of 2450 MHz EMF exposure in the pre- or postnatal periods on the development of female rats, decreased serum IGF1 levels were found in the postnatal exposure group, but not in the prenatal exposure group [55]. They also found increased levels of oxidative stress index and total oxidant status in the ovary tissues in both exposure groups, and stated that can be caused by the EMF induced chronic stress [55].

It is thought that the anti-angiogenic form of VEGF has an effect on survival and differentiation in germ cells, while the pro-angiogenic form is involved in self-renewal [56]. VEGF/VEGFR_2_ signaling has been shown to regulate germ cell proliferation and promote testicular regeneration through the enhancement of vascularization and direct action on germ cells [57]. In an in vitro study exposure to 2 mT, a 50 Hz magnetic field has shown to significantly reduce the expression and activation levels of VEGFR_2_, which is suggesting that magnetic field has a direct or indirect effect on VEGF receptors located on the cellular membrane [58]. In our study, we found that testis VEGF levels and immunoreactivity were decreased in the EMF-42 group. One study has suggested that the decrease in VEGF levels in diabetic rats is associated with an increase in apoptosis and testicular damage [59]. In another study, it has been shown that 15 Hz ELF-EMF stimulation reduces the renal expression of VEGF-A in diabetic rats [60]. In a study examining the effects of 2.45 GHz electromagnetic radiation on rat testicular tissue, it was found that the levels of MDA and total oxidant status increased, while the testosterone and VEGF levels did not change [61].

SHANK3 is a protein that increases actin polymerization and F-actin levels. A study, which showed the expression of SHANK2 and SHANK3 in testis, found that these proteins are associated with the acrosome in rat testicular germ cells [62]. SHANK2 and SHANK3 are thought to be able to bind the actin-based cytoskeleton of germ cells to the acrosomal membranes, as they are located close to the inner and outer membranes of the acrosome. It is thought that sperm acrosome reaction and neurotransmission might have some common mechanisms [62]. In SHANK3-deficient mice, loss of germinal cells in the seminiferous tubules, a few multinucleated giant cells, and empty epididymis associated with the affected testis were found, but it was stated that the spontaneous testicular degeneration may be specific to the strain [63]. In our study, the effect of perinatal EMF exposure, which was used as an environmental factor, on SHANK3 expression in the testis was examined for the first time. We found that in the group of EMF exposure up to postnatal 28th day decreased SHANK3 expression in testis, but not in the group of EMF exposure up to postnatal 42nd day. Although we think that this may be an adaptation of cells to environmental factors, it should be investigated in detail since there is not enough information in the literature about SHANK3 expression and its function in testis.

## 5. Conclusions

In conclusion, long-term EMF exposure during the pre- and postnatal periods may cause detrimental functional and histological effects alongside with the SHANK3 reduction on rat testes in late adolescence. EMF may affect the levels of SHANK3, IGF1, and VEGF, leading to structural changes and cause apoptosis, as we discovered when exposing embryos and adolescents to EMF. Our study supports that EMF may cause deterioration in testicular structure and function. We suggest that reproductive system disorders in men can be prevented by limiting the duration and strength of EMF exposure during the early developmental stages. In cases of unexplained subfertility and infertility, it should be considered that EMF exposure may cause damage during embryonic development and adolescence.

## Figures and Tables

**Table 1:** Body and organ weights of male rat pups (mean ± SEM).

Groups	*n*	Body Weight (g)	Testis Weight (mg)
Sham	15	144.14 ± 2.25	90.00 ± 7.02
EMF-28	10	140.32 ± 11.15	70.00 ± 12.39
EMF-42	10	115.44 ± 4.81 *	39.00 ± 3.77 *
*p* (Sham vs. EMF-42)		0.002	<0.001
*p* (EMF-28 vs. EMF-42)		0.013	0.020

* Significantly different from Sham and EMF-28 group (one-way ANOVA with subsequent LSD multiple comparisons test). EMF: 50 Hz electromagnetic field exposure (4 h/day, 5 days/week for 7 (EMF-28) and 9 (EMF-42) weeks); Sham group was exposed to the same conditions as the EMF group, except for the EMF exposure; *n*: number of rats; SEM: standard error of the mean.

**Table 2:** Sex hormone levels of male rat pups.

Hormones (Mean ± SEM)				
Groups	*n*	FSH (IU/L)	LH (mIU/mL)	Testosterone (ng/dL)
Sham	10	2.10 ± 0.22	5.52 ± 0.35	17.41 ± 3.25
EMF-28	10	2.25 ± 0.35	5.76 ± 0.12	25.81 ± 4.69
EMF-42	10	1.31 ± 0.15 *	4.73 ± 0.10 *	17.66 ± 3.38
*p* (Sham vs. EMF-42)		0.037	0.019	NS
*p* (EMF-28 vs. EMF-42)		0.015	0.003	NS

* Significantly different from Sham and EMF-28 group (one-way ANOVA with subsequent LSD multiple comparisons test). EMF: 50 Hz electromagnetic field exposure (4 h/day, 5 days/week for 7 (EMF-28) and 9 (EMF-42) weeks); Sham group was exposed to the same conditions as the EMF group, except for the EMF exposure; SEM: standard error of the mean; NS: not significant.

**Table 3:** MDA and GSH levels of male rat pups’ testis (mean ± SEM).

Group	*n*	MDA (µM/mg pr)	GSH (µM/mg pr)
Sham	15	0.55 ± 0.037	989.97 ± 66.98
EMF-28	10	0.69 ± 0.038 *	511.12 ± 75.53 ^#^
EMF-42	10	0.71 ± 0.038 *	959.89 ± 98.97
*p* (Sham vs. EMF-28)		0.014	<0.001
*p* (Sham vs. EMF-42)		0.006	NS
*p* (EMF-28 vs. EMF-42)		NS	0.001

* Significantly different from Sham group, ^#^ significantly different from Sham and EMF-42 group (one-way ANOVA with subsequent LSD multiple comparisons test). EMF: 50 Hz electromagnetic field exposure (4 h/day, 5 days/week for 7 (EMF-28) and 9 (EMF-42) weeks); Sham group was exposed to the same conditions as the EMF group, except for the EMF exposure; SEM: standard error of the mean; NS: not significant.

**Table 4:** SHANK3, IGF1 and VEGF levels of male rat pups’ testis (mean ± SEM).

Group	*n*	SHANK3 (ng/mL)	IGF1 (pg/mL)	VEGF (pg/mL)
Sham	15	11.64 ± 1.45	985.54 ± 41.17	331.66 ± 46.18
EMF-28	10	7.15 ± 0.87 ^#^	859.50 ± 72.23	238.55 ± 36.81
EMF-42	10	13.38 ± 0.96	737.51 ± 59.38 *	165.92 ± 34.08 *
*p* (Sham vs. EMF-28)		0.013	NS	NS
*p* (Sham vs. EMF-42)		NS	0.003	0.009
*p* (EMF-28 vs. EMF-42)		0.001	NS	NS

* Significantly different from Sham group, ^#^ significantly different from Sham and EMF-42 group (one-way ANOVA with subsequent LSD multiple comparisons test). EMF: 50 Hz electromagnetic field exposure (4 h/day, 5 days/week for 7 (EMF-28) and 9 (EMF-42) weeks); Sham group was exposed to the same conditions as the EMF group, except for the EMF exposure; SEM: standard error of the mean; NS: not significant.

## Data Availability

Not applicable.
